# Interactions between *Glu-1* and *Glu-3* loci and associations of selected molecular markers with quality traits in winter wheat (*Triticum aestivum* L.) DH lines

**DOI:** 10.1007/s13353-016-0362-5

**Published:** 2016-08-08

**Authors:** Karolina Krystkowiak, Monika Langner, Tadeusz Adamski, Bolesław P. Salmanowicz, Zygmunt Kaczmarek, Paweł Krajewski, Maria Surma

**Affiliations:** Institute of Plant Genetics, Polish Academy of Sciences, Strzeszyńska 34, 60-479 Poznań, Poland

**Keywords:** Genotype-by-environment interaction, *Glu-1* and *Glu-3* interaction, Hardness, Quantitative trait loci (QTL), Wheat

## Abstract

The quality of wheat depends on a large complex of genes and environmental factors. The objective of this study was to identify quantitative trait loci controlling technological quality traits and their stability across environments, and to assess the impact of interaction between alleles at loci *Glu-1* and *Glu-3* on grain quality. DH lines were evaluated in field experiments over a period of 4 years, and genotyped using simple sequence repeat markers. Lines were analysed for grain yield (GY), thousand grain weight (TGW), protein content (PC), starch content (SC), wet gluten content (WG), Zeleny sedimentation value (ZS), alveograph parameter W (APW), hectolitre weight (HW), and grain hardness (GH). A number of QTLs for these traits were identified in all chromosome groups. The *Glu-D1* locus influenced TGW, PC, SC, WG, ZS, APW, GH, while locus *Glu-B1* affected only PC, ZS, and WG. Most important marker-trait associations were found on chromosomes 1D and 5D. Significant effects of interaction between *Glu-1* and *Glu-3* loci on technological properties were recorded, and in all types of this interaction positive effects of *Glu-D1* locus on grain quality were observed, whereas effects of *Glu-B1* locus depended on alleles at *Glu-3* loci. Effects of *Glu-A3* and *Glu-D3* loci per se were not significant, while their interaction with alleles present at other loci encoding HMW and LMW were important. These results indicate that selection of wheat genotypes with predicted good bread-making properties should be based on the allelic composition both in *Glu-1* and *Glu-3 loci*, and confirm the predominant effect of *Glu-D1d* allele on technological properties of wheat grains.

**Electronic supplementary material:**

The online version of this article (doi:10.1007/s13353-016-0362-5) contains supplementary material, which is available to authorized users.

## Introduction

Common wheat (*Triticum aestivum* L.) is the second most globally important cereal crop after rice. The quality of wheat grain depends on many characteristics, among which protein compositions and grain hardness are the most important (Salmanowicz et al. 2008; Surma et al. 2012). Two major protein classes were identified: high molecular weight glutenins (HMW-GS) and low molecular weight glutenins (LMW-GS) (Bonafede et al. 2015). The HMW-GS are encoded by *Glu-A1, Glu-B1, Glu-D1* loci, (Goutam et al. 2013), whereas LMW-GS are encoded by a multi-gene family located at the *Glu-A3, Glu-B3*, and *Glu-D3* loci. High variation in allele composition has been detected both at *Glu-1* and *Glu-3* loci (Gale 2005). The allelic composition in *Glu-1* and *Glu-3* loci can be identified by the analysis of their protein products or directly by using functional markers (FMs) (Goutam et al. 2013; Liu et al. 2008). FMs can be used to test of wheat hybrids at early stages of breeding (Adamski et al. 2014). Genetic studies have shown that the subunit compositions of these proteins are correlated with bread-making quality (He et al. 2005; Luo et al. 2001), although the interrelations between HMW-GS and LMW-GS for quality performance are incompatible across experiments, mainly due to differences in the genetic background of the materials used (e.g., Jin et al. 2013; Liu et al. 2005). Overall, the gluten fraction explained 30–69 % of the variation in bread-making quality (Branlard et al. 2001), leaving a notable amount of variation determined by non-gluten factors, among others by kernel hardness.

Kernel hardness in wheat is controlled by the *Ha* locus on the short arm of chromosome 5D (Appels et al. 2001; Obuchowski et al. 2010). This locus is closely linked to loci *Pin-a* and *Pin-b*, encoding puroindolines, which are the main components of polypeptide friabilins. The wild alleles (alleles *Pina-D1a* and *Pinb-D1a*) are required together for soft wheat texture, and any mutation (including deletion), either in *Pina-D1* or *Pinb-D1* or both genes leads to increase of kernel hardness. Seventeen alleles of *Pina-D1* and 25 alleles of *Pinb-D1* in hexaploid wheat and the D genome of *Aegilops tauschii* were catalogued (http://www.shigen.nig.ac.jp/wheat/komugi/genes/symbolClassList.jsp). Alleles of *Pina-D1* and *Pinb-D1* can be identified in wheat plants using FMs (Liu et al. 2012). It was shown that marker for *Pinb-D1b* is most useful for wheat breeding because it is associated with good bread-making quality (Chen et al. 2012). Several studies have demonstrated that besides loci determining HMW-GS and LMW-GS, as well as grain hardness, also other regions of the wheat genome are responsible for grain technological traits. Analysis of quantitative trait loci (QTLs) based on genetic maps made it possible to detect genomic regions that control thousand grain weight (Groos et al. 2003), grain protein content (Prasad et al. 2003), sedimentation value (Li et al. 2009), starch content (Ma et al. 2005), kernel test weight (Sun et al. 2010), kernel hardness (Arbelbide and Bernardo 2006), wet gluten content (Li et al. 2009), and alveograph parameters (Zanetti et al. 2001), but molecular markers associated with technological properties are not effective in each breeding material. In the present studies, wheat doubled haploid (DH) lines derived from a cross between cultivars belonging to different quality classes were evaluated in terms of technological parameters. Objectives of our study were to (1) detect differences between parental cultivars and DH lines in molecular markers known to be associated with technological traits, (2) evaluate QTL effects and their stability across environments, and (3) assess the interaction between HMW-GS and LMW-GS and its influence on quality parameters.

## Materials and methods

### Plant materials

Materials for the studies consisted of doubled haploid (DH) lines of winter wheat derived from the Rysa x Finezja F_1_ hybrids by pollination with maize (Adamski et al. 2014). Parental cultivars belong to different bread quality classes: Finezja is a high-quality (A class) and Rysa is a medium quality (B class) and high-yielding, both cultivars bred in Poland. Parental cultivars and 120 DH lines were analysed previously in terms of alleles determining HMW-GS compositions. Rysa and Finezja possessed the same allele at *Glu-A1* locus (c), but differed at *Glu-B1* and *Glu-D1* loci: Rysa had *Glu-B1c* and *Glu-D1a*, Finezja had *Glu-B1b* and *Glu-d1d*. In the DH population, four classes of lines were distinguished according to alleles present at *Glu-B1* and *Glu-D1* loci. For present studies aimed at analysis of impact of HMW and LMW glutenins interaction on technological properties, we selected 60 lines, i.e., 15 lines out of each class, which constituted four subpopulations: G1: *Glu-B1b/Glu-D1a*, G2: *Glu-B1b/Glu-D1d*, G3: *Glu-B1c/Glu-D1d*, G4: *Glu-B1c/Glu-D1a*.

### Field experiments

Field experiments with 60 lines and parental cultivars were conducted during four seasons: 2010–2013. Each experiment was carried out in a randomised block design with three replicates. In each experiment, seeds were sown on 4 m^2^ plots with sowing density 300 seeds per 1 m^2^. The mean values for temperature and precipitation in May to July in the years 2010 to 2013 are presented in Table 1. Parents and DH lines were examined for their morphological and technological traits. After harvesting, thousand-grain weight (g) (TGW) and grain weight per plot (kg) (GY) were recorded.

**Table 1:** Meteorological data for years of experiment

Year	Sum of precipitation (in mm)			Mean temperature (in °C)		
	May	June	July	May	June	July
2010	110	26	84	11.5	17.4	22.1
2011	39	79	135	13.2	17.3	17.3
2012	23	144	64	15.0	17.8	20.2
2013	102	98	53	14.3	17.2	19.0

### Technological analysis

Grain protein content (%) (PC), starch content (%) (SC), alveograph parameter W (J) (APW) and hectoliter test weight (kg) (HW) and (%) (GH) were recorded using a Foss Infratec 1241 near-infrared-reflectance (NIR) Analyser (Foss, Hillerod, Denmark) (Silva et al. 2008). (The alveograph W parameter is a combination of dough strength and extensibility, and is expressed in joules.) Zeleny sedimentation value (ml) (ZS) was measured according to AACC method 56–61.02. Wet gluten content (%) (WG) was determined using AACC method 38–12. Data were recorded for each line in four environments.

### Isolation of HMW and LMW glutenin subunits and separation by SDS-PAGE

Extraction of HMW-GS from wheat flour was performed according to the method described by Salmanowicz (2008). LMW proteins were precipitated via the addition of 1-propanol to a final concentration of 85 % (*v/v*), and the samples were stored at 4 °C overnight. Before application to the gel protein, samples were dissociated by mixing in a 1:1 dissociating mixture and placing in a water bath at 100 °C for 5 min. HMW and LMW glutenin subunits were separated by electrophoresis in vertical SDS-PAGE gel on a Protean II xi cell unit (Bio-Rad, Hercules, CA, USA) using the discontinuous Tris–HCl-glycine buffer system of Laemmli (1970). Ten microliters of protein sample were separated at 240 V for 45 min after the tracking dye migrated off the gel. The gels were stained overnight with Coomassie Brilliant Blue G-250. The HMW-GS and LMW-GS bands were read as described by McIntosh et al. (2003 and supplement 2009).

### Functional marker analyses

Parental cultivars and DH lines were genotyped with gene-based markers for the loci *Glu–A1*, *Glu–B1*, *Glu–D1, Glu–A3*, *Glu–B3*, *Glu–D3*, *Pina–D1*, and *Pinb–D1*, according to the method of Liu et al. (2012). DNA was extracted following the methods described by Salmanowicz et al. (2012). After quantification of DNA concentration using a NanoDrop ND-1000 Spectrophotometer (NanoDrop Technologies, Wilmington, DE, USA), the samples were diluted in sterile water to 50 ng μl^−1^. For polymerase chain reaction (PCR) amplifications, a GeneAmp PCR system 9700 (Applied Biosystems, Life Technologies, Grand Island, NY, USA) was used.

### Microsatellite marker analyses

For genotyping, a total of 132 simple sequence repeats (SSR) loci were screened for polymorphisms between the parental genotypes. SSR markers were selected from previously published SSR-based genetic maps for wheat (Pestsova et al. 2000; Röder et al. 1998; Somers et al. 2004). Seven different sets of microsatellite primers were used: gwm (Röder et al. 1998), gdm (Pestsova et al. 2000), wmc (Wheat Microsatellite Consortium), and barc, cfa, cfd, and gpw (http://wheat.pw.usda.gov/GG2/index.shtml). SSR markers were chosen so that they corresponded to linkage groups 1A, 1B, 1D, 5A, 5B, and 5D, which are known to contain QTLs and candidate genes for bread quality traits (Arbelbide and Bernardo 2006; Perretant et al. 2000). In addition, SSRs associated with technological parameters were used for genotyping (Nelson et al. 2006; Mann et al. 2009; Kerfal et al. 2010; Reif et al. 2011).

DNA was extracted following the methods described by Salmanowicz et al. (2012). After quantification of DNA concentration using a NanoDrop ND-1000 Spectrophotometer (NanoDrop Technologies, Wilmington, DE, USA), the samples were diluted in sterile water to 50 ng μl − 1. For PCR amplification, a GeneAmp PCR system 9700 thermal cycler was used. Polymerase chain reactions were performed as described by Röder et al. (1998). Microsatellite alleles were detected using a 3130 Genetic Analyser (Applied Biosystems, Life Technologies, Grand Island, NY, USA).

### Statistical analysis

Two-way analysis of variance was carried out for each trait to determine the effects of environments (years) and genotypes, and of the genotype by environment interaction by the appropriate *F* statistics. The differences between means were verified by the least significant difference test (LSD) at *P* = 0.05. As a measure of the degree of linear association between two traits, the Pearson linear correlation coefficient for each pair of traits was calculated. The significance of the dependence of phenotypic traits on SSR marker genotypic classes was assessed using the *F*-test in analysis of variance (on a marker-by-marker basis, in the model including the marker by environment interaction effect). Environment-specific additive QTL effects at marker positions were calculated as half of the difference between the mean values for marker parental genotypic classes (Rysa–Finezja). Interaction between *Glu-1* and *Glu-3* loci was analysed by two-way analysis of variance. All computations were performed in Genstat 16 (VSN Int. 2013).

## Results

### HMW-GS and LMW-GS analysis

SDS-PAGE and analysis using gene-based markers for the loci *Glu-A1, Glu-B1, Glu-D1, Glu-A3, Glu-B3*, and *Glu-D3* revealed that parental cultivars differed in alleles at loci *Glu-B1* and *Glu-D1* (Rysa: *Glu-B1c* and *Glu-D1a;* Finezja: *Glu-B1b* and *Glu-D1d)*, whereas at the *Glu-A1* locus both cultivars had the same allele (*Glu-A1c*). Four groups of DHs representing all allele combinations at *Glu-B1* and *Glu-D1* loci were distinguished as mentioned in the “Materials and Methods” section. Within studied lines, eight different allele compositions at loci *Glu-A3, Glu-B3*, and *Glu-D3* determining LMW-GS were recorded: *Glu-A3b/Glu-B3a/Glu-D3c*; *Glu-A3e/Glu-B3d/Glu-D3g, Glu-A3b/Glu-B3a/Glu-D3g, Glu-A3b/Glu-B3d/Glu-D3c*, *Glu-A3b*/*Glu-B3d/Glu-D3g, Glu-A3e/Glu-B3a/Glu-D3c*, *Glu-A3e/Glu-B3a/Glu-D3g*, and *Glu-A3e/ Glu-B3d/Glu-D3c*. The compositions of HMW-GS and LMW-GS in DH lines and parental cultivars are shown in ESM 1.

### Puroindoline analysis

PCR products obtained for parental genotypes and DH lines indicated a lack of diversity at locus *Pina-D1* — both cultivars had the *Pina-D1a* allele. The results of analysis of alleles at the *Pinb-D1* locus are presented in ESM 1. Lines were classified as soft or medium on the basis of molecular analyses; in soft lines, wild alleles *Pina* and *Pinb* (*Pina-D1a*, *Pinb-D1a*) were identified, lines classified as medium had a mutated allele of *Pinb* (*Pinb-D1b*).

### Quality analysis of grain

In each year, a large differentiation among the lines was observed, and this was reflected in a large difference between extreme lines and in the coefficients of variation (Table 2). Great inter-individual differences measured as coefficients of variance (CV%) were found both for PC (8.71–24.19 %) and GH (19.32–23.67 %). The estimated mean values (ESM 1) and the ANOVA results for all of the analysed traits revealed significant (*P* < 0.01) variation between genotypes and years (Table 3). A significant genotype × environment (GE) interaction was also noted for all traits (Table 3). Mean value and coefficients of variation (CV%) in each subpopulation are presented in Table 4. The lowest PC, WG, ZS, APW, and GH values were observed for subpopulation G1 (*Glu-A1c/Glu-B1b/Glu-D1a*). The subpopulations of *Glu-D1d* lines (groups 2 and 3) were characterised by higher PC, WG, ZS, APW and GH values than *Glu-D1a* lines. Group 3 was distinguished by having the highest PC, WG, ZS, and APW values, but the SC value for this group was the lowest (Table 4). Group 2 was characterised by the highest GH value. High inter-individual differences measured as coefficients of variation (CV%) in subpopulations were found both for GY (8.39–10.37 %) and GH (10.72–16.98 %). Comparison of means for genotypes of medium (*Pina-D1a, Pinb-D1b*) and soft (*Pina-D1a, Pinb-D1a*) grains revealed that medium genotypes were characterised by significantly higher TGW, PC, WG, ZS, APW, and GH than soft lines. The differences between soft and medium groups were significant (*P* < 0.01) for all the analysed traits (Table 5).

**Table 2:** Means, ranges, and coefficient of variation (CV%) of analysed traits in a Rysa by Finezja DH lines wheat population using data from four years

Traits	Year	Parental lines		DH population				
		Rysa	Finezja	Minimum	Maximum	Mean	S.e.	CV^a^ %
GY (kg)	2010	1.70	1.87	0.70	1.90	1.29	0.04	24.19
	2011	1.01	1.27	0.72	1.33	1.07	0.02	12.73
	2012	2.59	1.13	1.13	2.73	1.99	0.05	18.97
	2013	1.91	1.91	1.57	2.53	2.02	0.02	8.71
TGW (g)	2010	41.13	41.13	32.95	43.73	38.83	0.31	6.24
	2011	46.86	45.4	40.28	50.91	45.76	0.29	4.95
	2012	41.98	40.55	32.86	45.75	39.06	0.34	6.79
	2013	48.08	46.25	37.73	51.20	46.21	0.34	5.72
PC (%)	2010	12.28	12.24	11.05	16.00	12.76	0.13	7.68
	2011	12.02	12.40	11.06	13.53	12.31	0.07	4.51
	2012	13.88	13.47	11.26	16.07	13.75	0.10	5.86
	2013	12.27	12.38	10.74	13.44	12.07	0.07	4.75
S C (%)	2010	68.14	69.02	65.64	69.92	68.09	0.12	1.41
	2011	69.21	69.18	66.84	70.29	68.83	0.09	1.02
	2012	67.76	67.67	65.06	69.80	67.45	0.12	1.42
	2013	67.67	69.87	67.30	70.96	69.24	0.11	1.24
WG (%)	2010	24.44	25.51	21.58	33.86	25.23	0.31	9.50
	2011	21.92	22.68	19.23	24.82	22.35	0.17	5.91
	2012	27.78	26.95	21.03	32.93	27.53	0.27	7.72
	2013	22.97	24.39	20.22	26.81	23.59	0.19	6.27
ZS (ml)	2010	33.51	33.05	22.36	54.15	36.61	0.86	18.23
	2011	30.48	32.58	26.92	37.38	31.95	0.27	6.65
	2012	40.56	32.05	27.36	55.86	39.56	0.73	14.20
	2013	36.60	34.51	23.85	41.99	33.42	0.46	10.75
APW (J)	2010	251.67	258.56	196.99	359.64	279.90	3.79	10.49
	2011	232.90	260.10	184.88	279.96	245.49	2.57	8.10
	2012	285.07	275.04	199.10	355.28	277.92	3.77	10.51
	2013	238.66	243.20	184.69	283.80	234.14	3.16	10.46
HW (kg)	2010	77.89	79.58	71.05	78.99	76.66	0.22	2.23
	2011	75.13	77.61	74.03	79.31	76.72	0.13	1.33
	2012	76.96	77.31	74.65	80.53	77.45	0.16	1.64
	2013	81.11	81.44	77.85	82.47	80.60	0.12	1.17
GH (%)	2010	17.06	32.13	18.92	54.93	37.30	1.09	22.73
	2011	21.79	26.38	6.84	36.28	23.89	0.73	23.67
	2012	21.63	32.36	15.90	42.46	29.86	0.74	19.32
	2013	21.26	37.88	16.11	54.20	29.63	0.86	22.40

^a^CV — coeffcient of variation computed for DH mean values

**Table 3:** Analysis of variance for analyzed traits of DH population

Source of variation	Degrees of freedom	GY		TGW		PC		SC		WG	
		Mean square	*F* statistic	Mean square	*F* statistic	Mean square	*F* statistic	Mean square	*F* statistic	Mean square	*F* statistic
Environments (E)	3	43.10	1436.67**	3036.88	2200.64**	101.48	507.4**	118.32	394.40**	926.92	785.53**
Genotypes (G)	61	0.29	9.67**	44.60	32.32**	2.86	14.30**	5.18	17.27**	16.69	14.14**
Interaction GE	183	0.21	7.00**	9.74	7.06**	1.23	7.17**	1.27	4.23**	8.19	6.94**
Error	496	0.03		1.38		0.20		0.30		1.18	
Source of variation	Degrees of freedom	ZS		APW		HW		GH			
		Mean square	*F* statistic	Mean square	*F* statistic	Mean square	*F* statistic	Mean square	*F* statistic		
Environments (E)	3	2111.85	348.49**	9634.54	43.75**	646.46	734.61**	5060.06	315.07**		
Genotypes (G)	61	103.14	17.02**	3804.57	17.28**	7.26	8.25**	291.05	17.72**		
Interaction GE	183	56.60	9.34**	1382.27	6.28**	4.06	4.61**	88.34	5.38**		
Error	496	6.06		220.22		0.88		16.42			

**Significant at *P* < 0.01

**Table 4:** Means and coefficients of variation (CV%) of analysed traits in four (G1–G4) subpopulations

Traits	G1: *Glu-B1b/Glu-D1a*		G2: *Glu-B1b/Glu-D1d*		G3: *Glu-B1c/Glu-D1d*		G4: *Glu-B1c/Glu-D1a*	
	Mean	CV (%)	Mean	CV (%)	Mean	CV (%)	Mean	CV (%)
GY (kg)	1.60	8.39	1.62	10.37	1.54	9.40	1.60	10.27
TGW (g)	42.59	4.85	42.32	3.71	41.56	5.03	43.41	3.91
PC (%)	12.40	3.24	12.77	4.16	13.07	3.22	12.65	3.27
SC (%)	68.73	0.66	68.34	0.87	68.05	1.12	68.49	0.99
WG (%)	23.86	4.28	24.90	4.78	25.49	4.41	24.45	3.64
ZS (ml)	33.38	8.05	35.67	8.59	37.14	5.88	35.35	8.19
APW (J)	250.2	5.39	265.0	6.93	268.4	6.64	253.8	6.79
HW (kg)	77.93	0.82	78.06	1.04	77.83	1.13	77.62	0.99
GH (%)	26.57	14.78	32.57	16.98	30.79	10.72	30.70	15.50

**Table 5:** Contrast analysis between groups of soft and medium lines

Traits								
GY	TGW	PC	SC	WG	ZS	APW	HW	GH
0.04**	−0.33**	−0.50**	0.70**	−1.24**	−3.31**	−20.70**	0.29**	−7.57**

^**^Significant at *P* < 0.01

Studied lines were characterised by relatively broad variation in grain yield per plot. Over the years, the mean value was equal to 1.60 kg; for the individual lines it ranged from 1.32 (line no. 19) to 1.96 kg (line no. 49). Over the years, the mean TGW value was equal to 42.51 g, and ranged from 37.12 g (line no. 36) to 47.04 g (line no. 51). Over the years, the mean value of protein content in grains of the studied genotypes ranged from 11.4 % (line no. 12) to 13.8 % (line nos. 22 and 38). General mean starch content amounted to 68.4 %, and ranged from 66.8 % to 69.9 % . Mean values over the 4 years of wet gluten obtained from the grain of studied genotypes ranged between 21.4 % and 28.0 %. Over the years, the mean Zeleny sedimentation values were recorded over a wide range for the studied lines, from 28.4 ml (line no. 12) to 41.9 ml (line no. 22). Mean values over the 4 years of the alveograph W parameter for particular genotypes ranged from 218.5 (line no. 49) to 302.7 J (line no. 38). Over the years, the mean HW value was equal to 77.9 kg, and ranged from 75.9 kg (line no. 56) to 79.6 kg (line no. 18) for the individual genotypes. Over the years, the mean GH value amounted to 30.00, and ranged from 17.2 to 44.5 for the individual lines (ESM 1).

### Correlations between traits

Strong associations were observed between grain technological properties (e.g., PC, WG, ZS, and APW) (Table 6). SC was negatively correlated with PC, WG, ZS, and APW, and positively correlated with HW, GY, and TGW. The correlations between GY and HW and other features were low and not significant. TGW was negatively correlated with PC, WG, ZS, APW, and GH, but correlation coefficients were not always significant. Strong positive associations with TGW were exclusively observed for GY.

**Table 6:** Correlation coefficients between grain technological parameters, grain yield and thousand-grain weight

	GY	TGW	PC	SC	WG	ZS	APW	HW
TGW	0.34**							
PC	0.01	−0.32						
SC	0.11	0.43**	−0.69**					
WG	−0.04	−0.32	0.95**	−0.58**				
ZS	−0.03	−0.36**	0.93**	−0.54**	0.93**			
APW	−0.17	−0.45**	0.85**	−0.70**	0.86**	0.64**		
HW	−0.04	0.07	−0.14	0.27	0.01	−0.12	0.04	
GH	−0.30	−0.66**	0.56**	−0.52**	0.64**	0.64**	0.63**	−0.02

^**^Significant at *P* < 0.01

### Microsatellite markers and QTL analysis

Seventy-one SSR markers out of 132 were found to be polymorphic in the parental genotypes. Marker Xgpw4002 was closely linked with the *Glu-B1* locus. The 208-bp allele was observed in all lines with alleles *Glu-B1c*, whereas the 211-bp allele was observed in lines *Glu-B1b*.

QTLs expressing significant mean effect or significant interaction with years (*P* < 0.001) were detected for all of the observed traits (ESM 2). Out of 120 significant marker-trait associations found, the largest number of QTLs was noted for TGW (20 QTLs), and the smallest number – for HW (two QTLs). Interaction of the allelic effect with environment characterized 26.7 % of QTLs; this interaction was most frequent for GY (five out of seven QTLs) and ZS (11 out of 17 QTLs). On the other hand, the QTL effects were mostly stable over environments for TGW (lack of interaction for 19 out of 20 QTLs). Within years, the frequency of significant QTL effects was the largest in 2010 (86.7 %, with the majority—50.8 %—being positive), and the smallest in 2013 (46.7 %, with the majority—33.3 %—being negative). The mean over-years QTL effects were most frequently positive for ZS (13 out of 17 QTLs).

### Grain yield

A significant and stable (no interaction with years) QTL effect for GY was noted at markers Xwmc407 and Xgwm635. For Xwmc407, located in chromosome 2A, a positive effect on GY was attributed to the Rysa allele. For Xgwm635 (located in 7D), a positive effect was attributed to the Finezja allele. The interaction of QTL effects with years for this trait was revealed for five markers (Xwmc312, Xwmc336, Xgwm131, Xbarc061, and Xwmc419): statistically different additive effects were observed in different years.

### Thousand grain weight

A significant QTL effect on TGW was found for 20 molecular markers. The markers of greatest and most significant effect in all years were localised on chromosomes 1A, 4A, and 7A (Xwmc93, Xgwm160, Xgwm63). Interaction with year was revealed for one molecular marker, Xbarc 130 on chromosome 5D, for which positive or negative effects were observed in different years.

### Protein content

Sixteen marker–trait associations in nine chromosomes were identified for protein content. QTLs of greatest and most significant effects in all years were localised on chromosomes 5A and 1D (associated with Xbarc130 and *Glu-D1*). At both of these loci, the Finezja allele had a positive effect. The highest additive effects for these markers were detected in 2010. The marker × year interaction was significant for seven molecular markers. Only one marker (Xgwm273) had a different sign for the additive effects in different years. For most of these markers (*Glu-B1*, Xgpw4002, Xwmc474 and Xwmc 344), the highest additive effect was observed in 2010, and the lowest in 2013.

### Starch content

Thirteen significant QTL effects for SC were identified in seven chromosomes. The major QTL was identified on chromosome 5D at Xbarc130, with the additive effects significant in all years, and the Rysa allele increasing starch content. Marker genotype × year interaction was only recorded for Xwmc 134 and Xwmc 344. The additive effects of these markers were significant only in 2 years (2010 and 2013 as well as 2010 and 2011 for Xwmc134 and Xwmc344 respectively). In both cases, the Finezja allele had a positive effect on SC.

### Wet gluten content

For wet gluten, 18 marker–trait associations were identified. The largest QTL effect was identified on chromosome 5D, and was associated with the Xbarc130 marker (5D). The additive effects of this marker were significant in all years, and the Finezja allele increased WG content. Significant additive effects were identified in all years for markers *Glu-D1* (1D) and Xgdm126 (5A). In both cases, the Finezja allele had a positive influence on WG. A significant interaction (*P* < 0.001) of QTL effects with the year was noted for four markers. The additive effects of the markers *Glu-B1*, Xwmc134, and Xgpw4002 were positive in all years; however, they were significant only in 2010 and 2011, while for the Xgwm273 marker, the additive effects were of different sign in different years.

### Zeleny sedimentation value

Seventeen molecular markers were associated with ZS. The QTL of greatest and most significant effect in the 4 years was localised on chromosome 5D, and was associated with marker Xbarc130. At this QTL, the Finezja allele had a positive effect. A significant interaction with years, as a consequence of the different additive effects (positive or negative) observed in different years, was noted for five markers (Xgwm273, Xwmc93, Xbarc061, gdm36, and Xwmc419). A significant interaction with years for six molecular markers (*Glu-B1*, *Glu-D1*, Xwmc134, Xgpw4002, Xwmc474, Xwmc344) was observed—the additive effects for these markers were significant only in 1 or 2 years.

### Alveograph W parameter

Twelve marker–trait associations were identified for APW on seven different chromosomes (1A, 1B, 1D, 2A, 2B, 5A, and 5D). The largest QTL effects were identified at Xbarc130 on chromosome 5D and *Glu-D1* on chromosome 1D. The additive effects at these markers were significant in all years, and the highest effect was observed in 2010. In both cases, the Finezja allele had a positive effect on APW. A significant interaction with years was found only for one molecular marker (Xwmc134) on chromosome 1B. The highest additive effect for this marker was observed in 2010, and the lowest was in 2012.

### Hectolitre weight

One significant mean QTL effect for HW was identified on chromosome 3B, with the Finezja allele having a positive influence. A QTL with effect interacting with environment was observed at marker Xgpw2276 on 1A as a consequence of different signs of the additive effects in different years.

### Grain hardness

Fifteen marker–trait associations for GH were identified. The most important marker–trait association was located on chromosome 7D (Xgwm111), and indicated that the Rysa allele had a significant positive effect over the 4 years. A significant interaction with the years (positive or negative effects in different years) was noted for the three markers on 1B, 1A, and 2B (Xwmc134, Xwmc93, Xwmc344).

### Interaction between HMW-GS and LMW-GS

Results presented in this study indicate significant interactions *Glu-1 * × *Glu-3*, *Glu-1 * × *Glu-1*, or *Glu-3 * × *Glu-3* for all the analysed traits, except TGW, although for this trait significant additive effects of *Glu-D1* and *Glu-B3* were detected (Table 7, ESM 2). Interestingly, *Glu-A3* and *Glu-D3* loci interacted with some other *Glu-1* and *Glu-3* loci, although additive effects of *Glu-A3* and *Glu-D3* were not significant for any traits. Significant effects of *Glu-D1* x *Glu-B1*, *GluD1* x *Glu-A3* and *Glu-D1* x *Glu-D3* interactions on studied parameters were revealed. It can be seen that, in these situations, the allele *Glu-D1d* from Finezja caused an increase in PC, WG, ZS, APW, and GH (Table 7). For GY the significant interaction effect was recorded only for *Glu-D1 * × *Glu-D3*. The *Glu-D3c* allele decreased GY in the presence of *Glu-D1a*, but increased GY in the presence of *Glu-D1d*. Interactions between all the analysed *Glu-1* and *Glu-3* loci were observed for PC, ZS, and APW. It was found that influence of *Glu-D3c* on PC was dependent on the *Glu-B1* allele, but this influence was not the same in each case, since both increase and decrease of PC values were observed. Similar interactions were observed for *Glu-B1* and *Glu-D3*; the *Glu-D3c* allele increased the value of APW and ZS in the presence of *Glu-B1c* but decreased it in the presence of *Glu-B1b*. For the GH interaction, *Glu-A3* × *GluB3* and *GluB1*  × *Glu-D1* were significant, although additive effects of QTL was detected only in *Glu-D1*. Interaction between *Glu-B3* and *Glu-D3* loci was significant for HW; however, additive effect of *Glu-3* loci for that trait was not detected.

**Table 7:** Interactions between genotypes in HMW-GS and LMW-GS markers with respect to influence on observed traits (ANOVA, significant at *P* < 0.01)

Trait	Maker 1	Marker 2	*P*-value for interaction	Mean values in genotypic classes (marker 1, marker 2)				Std. error of mean value
				A, A	A, B	B, A	B, B	
GY	*Glu-D1*	*Glu-D3*	0.002	1.644	1.550	1.492	1.642	0.04
TGW	*-*	*-*	-	-	-	-	-	-
PC	*Glu-A3*	*Glu-D1*	<0.001	12.31	13.09	12.72	12.82	0.07
	*Glu-B1*	*Glu-B3*	0.008	13.14	12.72	12.59	12.59	0.08
	*Glu-B1*	*Glu-D3*	<0.001	12.63	13.06	12.68	12.50	0.08
SC	*Glu-B1*	*Glu-B3*	<0.001	67.84	68.48	68.51	68.55	0.09
WG	*Glu-A3*	*Glu-D1*	0.002	23.67	25.47	24.58	25.04	0.21
	*Glu-B1*	*Glu-D3*	<0.001	24.38	25.49	24.66	24.13	0.21
ZS	*Glu-A3*	*Glu-B3*	0.007	36.12	33.38	36.04	35.81	0.48
	*Glu-A3*	*Glu-D1*	<0.001	32.83	37.05	35.70	36.02	0.43
	*Glu-B1*	*Glu-D3*	<0.001	34.87	37.45	34.97	34.13	0.43
APW	*Glu-A3*	*Glu-D1*	<0.001	245.1	273.3	258.1	262.9	2.50
	*Glu-B1*	*Glu-B3*	0.005	270.0	256.7	256.5	258.3	2.71
	*Glu-B1*	*Glu-D3*	0.004	253.6	267.6	258.1	257.2	2.57
	*Glu-B3*	*Glu-D3*	0.009	253.7	268.9	256.9	258.0	2.70
HW	*Glu-B3*	*Glu-D3*	0.009	77.56	78.07	78.05	77.67	0.17
GH	*Glu-A3*	*Glu-B3*	0.004	30.66	28.08	29.78	31.12	0.67
	*Glu-B1*	*Glu-D1*	<0.001	30.70	30.79	26.57	32.55	0.62

A- Rysa alleles

*Glu-B1* *c*

*Glu-D1a*

*Glu-A3e*

*Glu-B3d*

*Glu-D3g*

B- Finezja alleles

*Glu-B1* *b*

*Glu-D1d*

*Glu-A3b*

*Glu-B3a*

*Glu-D3c*

## Discussion

The results of the present study show that all the analysed traits were greatly influenced by genotype, environment, and GE interactions. The large variation of lines observed in each environment, with relatively small differences between parents, suggests the dispersion of genes controlling quality traits along parental genomes. Our results confirm the quantitative nature of quality parameters, which has been frequently reported in the literature (e.g., Huang et al. 2006; Krystkowiak et al. 2009; Sun et al. 2010).

Yong et al. (2004) found that grain hardness and sedimentation values were primarily influenced by genetic factors, whereas thousand kernel weight and test weight were affected by environments. Surma et al. (2012) also demonstrated that genotype has a greater influence on kernel hardness than environment. On the other hand, many other studies have demonstrated that environmental conditions have a larger effect on protein content than the genetic background (Mut et al. 2010). Surma et al. (2012) has also shown that the alveograph parameter was influenced to a similar degree by genotype and environment. Budak et al. (2003) and Tianu et al. (1996) demonstrated that GE has a small effect on sedimentation. Tianu et al. (1996) has also shown that the environmental influence was greatest on wet gluten content.

The phenotypic values of Finezja were higher than those of Rysa for most analysed traits, except for Zeleny sedimentation, grain yield, and thousand grain weight. However, both parents contributed positive alleles for all traits (ESM 2). In the present study, one of six QTLs for grain yield, 13 QTLs for thousand grain weight, seven of the 16 QTLs for protein content, four QTLs for starch content, four QTLs for alveograph parameter W, two QTLs for hectolitre weight, nine of 18 QTLs for wet gluten, one of 17 QTLs for Zeleny sedimentation value, and eight QTLs for grain hardness, were detected in at least three environments. However, for a large number of QTLs a significant interaction with environment was detected; 59 % QTLs were detected only in one or two environments, so they cannot be considered as “stable” QTLs, but as environment-dependent. These results confirm the need to perform trials in multiple environments for precise evaluation of QTL effects.

Both grain yield and thousand grain weight can be affected by genetic factors, environments, and genotype–environment interactions. For grain yield, no QTLs stable in all environments were identified. TGW is one of the most important yield components and is correlated with technological parameters, as reported, e.g., by Zanetti et al. (2001). In our study, three thousand grain weight QTLs on chromosomes 1A, 4A, and 7A were stable across all environments, making them prime candidates for marker-assisted selection. Positive alleles of two of these QTLs originated from Rysa. In contrast, Groos et al. (2003) identified three QTLs for thousand grain weight on chromosomes 2B, 5B, and 7A in all environments. Ramya et al. (2010) identified ten QTLs for thousand grain weight on chromosomes 1A, 1D, 2B, 2D, 4B, 5B, and 6B. Seventeen QTLs for TGW, covering all groups of chromosomes except for group 3, were detected by Elangovan et al. (2011).

Grain protein content is a quantitative trait controlled by several genes distributed throughout the wheat genome (Groos et al. 2003; Huang et al. 2006; Prasad et al. 2003). In our study, QTLs of the major effects were detected on chromosomes 5D (Xbarc130) and 1D (*Glu-D1*). These results are in agreement with the QTL localisation previously reported by Nelson et al. (2006) and Zanetti et al. (2001). These authors revealed QTLs for protein content to the storage protein loci on chromosomes of group 1 — *Glu-1* and *Glu-3*. In our experiment, two protein content QTLs on chromosomes 5A and 5D (linked to markers gdm126 and Xbarc130 respectively) were stable across all environments, making them prime candidates for marker-assisted selection. These results are in agreement with the QTL localisation previously reported by Weightman et al. (2008) and Li et al. (2009). A total of 13 QTLs for protein content in individual environments, spread on eight chromosomes, were detected by Prasad et al. (2003), but only five of these QTLs were considered to be important; four of these QTLs (on chromosome 2B, 2D, 3D, and 7A) were identified in more than one location, and one QTL (3D) was identified using means for all the environments.

Only one QTL for starch content stable in four environments was detected in our study, on chromosome 5D, and an allele contributed by cv. Rysa increased starch content. Nine QTLs were detected only in one or two environments, but significant interactions (*P* < 0.001) with year were observed for only two QTLs. QTLs for starch content were found on chromosomes 1, 2, and 5 homologous groups, and these results are similar to those of Reif et al. (2011). These authors found three QTLs for starch content on chromosomes 5A, 5B, and 1D.

We also identified the twelve QTLs for alveograph parameter W, which were localised on chromosomes of homologous groups 1, 2, and 5. One of these QTLs with the largest effect was on chromosome 1D at the locus *Glu-D1*. These results are consistent with several other studies, in which QTLs for dough strength were identified on chromosomes 1A, 1B, 1D, 2A, 2D, 5A, 5B, and 5D (Crepieux et al. 2005; Kerfal et al. 2010; Zanetti et al. 2001). Moreover, Nelson et al. (2006) found QTL for alveograph L at the locus *Glu-A3* and QTL for alveograph W associated with the locus *Gli-B1*. On the other hand, Perretant et al. (2000) noted that alveograph parameter W was associated with the locus *Glu-A1*.

Eighteen QTLs for wet gluten were localised on chromosomes in homologous group 1, 2, 5, and 7. Two of these QTLs on chromosomes 1D and 5A were stable in all environments. The interaction of marker effects with environment was significant (*P* < 0.001) for four QTLs. Similarly, Li et al. (2009) detected 16 QTLs for wet gluten on chromosomes of all homologous groups, but only five of them were identified in more than one environment.

The largest QTL affecting Zeleny sedimentation value was located on chromosome 5D, and its effect was significant in all years. Our result is consistent with several other studies, in which QTLs for sedimentation were reported on chromosome 5D (Huang et al. 2006; Li et al. 2009) and 5B (Kerfal et al. 2010; Zanetti et al. 2001). In our study, we also detected QTLs on 1B, 1D, 2B, 3A, and 7B, but their effects were not significant in all years. Huang et al. (2006) and McCartney et al. (2006) have identified the most important QTL for sedimentation on chromosome 1B. In contrast to their results, Blanco et al. (1998) and Kerfal et al. (2010) identified QTLs for sedimentation in the homologous group 7.

In this study, the most important marker–trait association for grain hardness was located on chromosome 7D (ESM 2). The most stable QTLs detected in all environments were located on chromosomes 1D (linked to marker Xgwm106) and 7D (linked to marker Xgwm111). Thus, these markers may prove to be most valuable in breeding programmes for the improvement of wheat quality. Previous studies have indicated that chromosome 5D is an important factor that affects grain hardness; thus, QTLs for grain hardness are often distally localised on chromosome 5D (Li et al. 2009; Mann et al. 2009; Nelson et al. 2006; Sun et al. 2010). In our study, QTL for grain hardness was also located on chromosome 5D (associated with marker Xbarc130), but the interaction of its effect with environments was significant *(P* < 0.001). In the present study, other QTLs were also located on homologous groups 1, 2, 4, and 7, but seven of these were detected only in one or two environments. The interaction of allelic effect with environments was significant (*P* < 0.001) for four QTLs (on 1A, 1B, 2B ,and 5D). This finding, as well as results from other studies, indicate that the *Ha* locus is important for grain hardness but that grain hardness is influenced also by other loci located on all wheat chromosomes (Groos et al. 2004; Li et al. 2012; Tsilo et al. 2011; Weightman et al. 2008). Our results indicate that selection of wheat for good technological parameters that is only based on molecular markers connected with the *Ha* locus may be ineffective.

The present study provides useful information on the interactions between *Glu-1* and *Glu-3* loci and their influence on technological properties. It is known that both HMW-GS and LMW-GS play important roles in determining the quality of wheat, so several studies have been focused on the effects of allelic variation at both the *Glu-1* and *Glu-3* loci. Generally, allelic variation in the *Glu-1* had a greater effect on quality than *Glu-3* (Rodriguez-Quijano et al. 2001). *Glu-D1* locus has the largest effect on the quality of wheat, followed by the *Glu-B1* and *Glu-A1* loci. Moreover, several authors have demonstrated larger effects of *Glu-D1d* loci on baking quality compared with *Glu-D1a*. In addition, these authors have also demonstrated a positive impact of *Glu-D1d* on sedimentation, dough strength, and mixing time (Dobraszczyk et al. 2005; He et al. 2005; Liang et al. 2010). These results are consisted with the results of our study; the subpopulations possessing different *Glu-D1* alleles [G2 and G3 (*Glu-D1d*), G1 and G4 (*Glu-D1a*)] differed in all of the analysed traits. Lines with *Glu-D1d* showed higher values of PC, WG, ZS, APW, and GH than lines with alleles *Glu-D1a*. Other authors demonstrated that *Glu-B1b* genotypes were observed to have higher sedimentation values than *Glu-B1c*. (He *et al*. 2005; Liu et al. 2005), but opposite results were obtained by Huang et al. (2006) and Ram (2003). Jin et al. (2013) indicated that alleles *Glu-A3e*, *Glu-B3b* and *Glu-D3c* positively influenced baking quality of wheat. In addition, He et al. (2005) demonstrated that the positive contribution of allele *d* at the *Glu-B3d* locus is higher than the contributions of *b* and *f* to farinograph stability.

The interaction between *Glu-1* and *Glu-3* loci has also been confirmed in several previous studies (Branlard et al. 2001; Jin et al. 2013; Liu et al. 2005; Ma et al. 2005), but analysis of interactions between these loci with respect to its influence on technological properties has not been studied in detail. The present study indicated that *Glu-1* may significantly modify effects of *Glu-3* alleles, just as *Glu-3* influences the effect of *Glu-1*. This finding may be a particularly important addition to the QTL analysis. Additive effects at *Glu-D1* were detected for all traits except grain weight and hectolitre weight, but additive effects at *Glu-B1* and *Glu-B3* were detected only for three and two traits respectively. Interestingly, both *Glu-B1* and/or *Glu-B3* interacted with other loci with respect to all traits except grain yield. Moreover, no significant additive effects at *Glu-A3* and *Glu-D3* loci were found; however, based on the interaction between *Glu-1* and *Glu-3* loci, we indicated that effects of these loci could be important.

This finding suggests that breeders can select—within breeding lines of disadvantageous HMW-GS composition—genotypes that possess favourable traits for LMW glutenin subunits and in this way improve the technological properties of wheat grains.

## Conclusions

The *Glu-D1* locus influenced seven out of the nine analysed traits, i.e., TGW, PC, SC, WG, ZS, APW, and GH, while locus *Glu-B1* affected only three traits—PC, ZS, and WG . Most important marker–trait associations were found on chromosomes 1D and 5D for SSR markers (Xwmc336, Xwmc36, Xwmc732, Xgpw315, Xgwm 106, Xgwm 642, and Xbarc130) Significant effects of interaction between *Glu-1* and *Glu-3* loci on technological properties were recorded, and in all types of this interaction stable and positive effects of *Glu-D1* loci on grain quality were observed, whereas effects of *Glu-B1* locus depended on alleles at *Glu-3* loci. Effects of *Glu-A3* and *Glu-D3* loci per se were not significant, while their interaction with alleles presented at other loci encoding HMW and LMW was important. Our results indicate that selection of wheat genotypes with predicted good bread-making properties should be based on composition of both HMW and LMW subunits, and confirm the predominant effect of *Glu-D1d* allele encoding 5 + 10 HMW subunits on technological properties of wheat grains.

## Electronic supplementary material

Below is the link to the electronic supplementary material.

ESM 1 (DOCX 90 kb)

ESM 2 (DOCX 65 kb)
