# Supplementary material for: Interactions between Glu-1 and Glu-3 loci and associations of selected molecular markers with quality traits in winter wheat (Triticum aestivum L.) DH lines
Source: J Appl Genet. 2016 Aug 8;58(1):37–48. doi: 10.1007/s13353-016-0362-5 (PMC5243893; doi:10.1007/s13353-016-0362-5)
Supplement: Supplementary file 1 — (DOCX 90 kb) [file 13353_2016_362_MOESM1_ESM.docx]

Interactions between Glu-1 and Glu-3 loci and associations of selected molecular markers with quality traits in winter wheat (*Triticum aestivum* L.) DH lines

Krystkowiak Karolina*, Langner Monika*, Adamski Tadeusz, Salmanowicz Bolesław, Kaczmarek Zygmunt, Krajewski Paweł, Surma Maria

Institute of Plant Genetics, Polish Academy of Sciences, Strzeszyńska 34, 60-479 Poznań, Poland

* E-mail: kkry@igr.poznan.pl

mdyl@igr.poznan.pl

ESM_1. Over years mean values of analyzed grain technological traits and allele composition in *Pin-D1*, *Glu-1* and *Glu-3* loci

| Line | | GY | TGW | PC | SC | WG | ZS | APW | HW | GH | *Pinb-D1* | *GluA1/Glu-B1/Glu-D1* | *Glu-A3/Glu-B3/Glu-D3* |
| --- | --- | --- | --- | --- | --- | --- | --- | --- | --- | --- | --- | --- | --- |
| 1 |  | 1.41 | 40.87 | 12.2 | 68.8 | 23.2 | 31.4 | 246.2 | 77.0 | 24.6 | *a* | *c/b/a* | *b/a/c* |
| 2 |  | 1.56 | 45.28 | 12.8 | 68.2 | 24.6 | 36.0 | 269.6 | 77.7 | 33.3 | *b* | *c/b/a* | *b/a/c* |
| 3 |  | 1.86 | 43.65 | 12.4 | 69.0 | 23.7 | 33.7 | 247.5 | 78.0 | 24.2 | *a* | *c/b/a* | *b/a/c* |
| 4 |  | 1.67 | 42.03 | 12.5 | 67.9 | 23.8 | 33.4 | 267.6 | 78.3 | 31.0 | *b* | *c/b/a* | *b/a/c* |
| 5 |  | 1.65 | 41.91 | 12.2 | 68.9 | 22.9 | 30.8 | 229.3 | 77.1 | 24.6 | *a* | *c/b/a* | *e/d/g* |
| 6 |  | 1.64 | 45.17 | 12.4 | 68.5 | 24.2 | 35.3 | 246.3 | 77.8 | 30.2 | *b* | *c/b/a* | *e/d/g* |
| 7 |  | 1.62 | 45.87 | 12.9 | 68.6 | 24.9 | 36.7 | 254.9 | 77.8 | 28.9 | *a* | *c/b/a* | *b/a/g* |
| 8 |  | 1.48 | 43.06 | 12.8 | 69.1 | 25.2 | 36.1 | 262.4 | 78.6 | 29.2 | *a* | *c/b/a* | *b/a/g* |
| 9 |  | 1.56 | 40.22 | 12.7 | 68.3 | 24.9 | 36.0 | 264.8 | 78.4 | 29.0 | *a* | *c/b/a* | *b/d/c* |
| 10 |  | 1.34 | 43.44 | 12.7 | 69.2 | 24.8 | 36.2 | 247.1 | 76.9 | 25.4 | *a* | *c/b/a* | *b/d/c* |
| 11 |  | 1.64 | 41.00 | 12.6 | 68.2 | 24.3 | 33.4 | 256.9 | 78.7 | 25.3 | *a* | *c/b/a* | *b/d/g* |
| 12 |  | 1.50 | 40.11 | 11.4 | 68.9 | 21.4 | 28.4 | 222.2 | 77.3 | 23.1 | *a* | *c/b/a* | *e/a/c* |
| 13 |  | 1.78 | 40.19 | 12.1 | 68.6 | 23.1 | 30.9 | 255.1 | 78.4 | 25.2 | *a* | *c/b/a* | *e/a/c* |
| 14 |  | 1.61 | 40.88 | 12.0 | 69.4 | 22.9 | 29.5 | 243.3 | 78.0 | 17.2 | *a* | *c/b/a* | *e/a/g* |
| 15 |  | 1.68 | 45.09 | 12.3 | 69.4 | 24.0 | 32.8 | 240.4 | 78.9 | 27.4 | *a* | *c/b/a* | *e/a/g* |
| 16 |  | 1.70 | 40.35 | 12.8 | 68.5 | 24.7 | 35.0 | 260.4 | 77.8 | 28.5 | *a* | *c/b/d* | *b/a/c* |
| 17 |  | 1.72 | 43.75 | 13.0 | 68.2 | 25.3 | 35.7 | 270.9 | 77.9 | 26.8 | *a* | *c/b/d* | *b/a/g* |
| 18 |  | 1.34 | 40.43 | 11.9 | 69.9 | 23.1 | 30.7 | 223.2 | 79.6 | 25.0 | *a* | *c/b/d* | *b/a/g* |
| 19 |  | 1.32 | 40.05 | 12.7 | 68.0 | 24.9 | 36.0 | 270.7 | 77.2 | 36.6 | *b* | *c/b/d* | *b/a/g* |
| 20 |  | 1.56 | 42.29 | 12.7 | 68.5 | 25.6 | 36.6 | 278.4 | 78.6 | 44.5 | *b* | *c/b/d* | *b/a/g* |
| 21 |  | 1.54 | 44.10 | 12.8 | 68.1 | 24.9 | 35.8 | 256.6 | 78.0 | 35.6 | *b* | *c/b/d* | *b/a/g* |
| 22 |  | 1.37 | 43.20 | 13.8 | 67.4 | 26.9 | 41.9 | 287.4 | 78.3 | 37.4 | *b* | *c/b/d* | *b/a/g* |
| 23 |  | 1.79 | 43.10 | 13.0 | 68.4 | 25.8 | 38.8 | 269.3 | 78.9 | 37.1 | *b* | *c/b/d* | *b/d/c* |
| 24 |  | 1.55 | 40.16 | 13.1 | 68.2 | 25.7 | 37.1 | 271.8 | 76.8 | 29.6 | *b* | *c/b/d* | *b/d/c* |
| 25 |  | 1.73 | 41.87 | 11.7 | 68.6 | 22.1 | 28.9 | 230.2 | 78.0 | 23.1 | *a* | *c/b/d* | *b/d/c* |
| 26 |  | 1.80 | 41.59 | 12.4 | 68.5 | 24.0 | 34.2 | 263.4 | 77.4 | 32.2 | *b* | *c/b/d* | *b/d/c* |
| 27 |  | 1.77 | 42.91 | 13.5 | 67.4 | 26.2 | 38.1 | 293.1 | 76.8 | 32.3 | *b* | *c/b/d* | *e/a/g* |
| 28 |  | 1.71 | 44.14 | 12.6 | 68.8 | 24.5 | 35.0 | 260.4 | 78.0 | 31.0 | *b* | *c/b/d* | *e/d/c* |
| 29 |  | 1.67 | 42.01 | 12.7 | 68.3 | 24.9 | 35.4 | 270.1 | 78.6 | 34.2 | *b* | *c/b/d* | *e/d/c* |
| 30 |  | 1.76 | 44.81 | 12.9 | 68.3 | 25.1 | 35.7 | 268.9 | 79.2 | 31.8 | *b* | *c/b/d* | *e/d/c* |
| 31 |  | 1.38 | 40.70 | 13.1 | 67.4 | 26.0 | 38.7 | 276.5 | 78.3 | 36.9 | *b* | *c/c/d* | *b/a/c* |
| 32 |  | 1.61 | 44.98 | 12.9 | 69.3 | 25.5 | 36.4 | 262.4 | 78.7 | 29.6 | *a* | *c/c/d* | *b/a/c* |
| 33 |  | 1.68 | 40.56 | 12.7 | 68.2 | 24.4 | 34.0 | 270.4 | 78.0 | 28.7 | *a* | *c/c/d* | *b/a/c* |
| 34 |  | 1.43 | 42.49 | 13.1 | 68.4 | 25.4 | 38.9 | 254.5 | 77.3 | 30.9 | *b* | *c/c/d* | *b/a/c* |
| 35 |  | 1.60 | 42.79 | 13.0 | 67.5 | 24.3 | 35.0 | 243.2 | 76.2 | 30.6 | *b* | *c/c/d* | *b/a/c* |
| 36 |  | 1.34 | 37.12 | 13.5 | 66.8 | 25.9 | 38.6 | 273.0 | 76.3 | 34.3 | *b* | *c/c/d* | *e/d/g* |
| 37 |  | 1.48 | 43.71 | 12.4 | 68.7 | 24.6 | 34.6 | 253.4 | 77.6 | 31.1 | *b* | *c/c/d* | *b/a/g* |
| 38 |  | 1.61 | 41.00 | 13.8 | 67.3 | 28.0 | 40.2 | 302.7 | 78.2 | 35.3 | *b* | *c/c/d* | *b/d/c* |
| 39 |  | 1.40 | 39.36 | 12.8 | 67.6 | 24.8 | 36.1 | 248.8 | 77.1 | 29.7 | *a* | *c/c/d* | *b/d/g* |
| 40 |  | 1.71 | 43.69 | 13.0 | 68.8 | 25.7 | 37.4 | 257.3 | 79.0 | 28.5 | *a* | *c/c/d* | *e/a/c* |
| 41 |  | 1.53 | 40.50 | 12.4 | 69.0 | 23.7 | 33.6 | 255.1 | 79.0 | 24.8 | *a* | *c/c/d* | *e/a/g* |
| 42 |  | 1.79 | 41.43 | 13.0 | 68.3 | 24.9 | 36.4 | 265.2 | 77.8 | 26.6 | *a* | *c/c/d* | *e/d/c* |
| 43 |  | 1.53 | 42.48 | 13.4 | 68.0 | 25.7 | 37.9 | 282.0 | 77.3 | 31.2 | *b* | *c/c/d* | *e/d/c* |
| 44 |  | 1.34 | 39.13 | 13.6 | 66.9 | 26.9 | 39.4 | 301.0 | 78.7 | 34.6 | *b* | *c/c/d* | *e/d/c* |
| 45 |  | 1.72 | 43.41 | 13.4 | 68.5 | 26.6 | 40.1 | 280.7 | 77.9 | 29.2 | *b* | *c/c/d* | *e/d/c* |
| 46 |  | 1.46 | 44.49 | 13.2 | 68.3 | 25.2 | 37.9 | 272.9 | 77.1 | 34.8 | *b* | *c/c/a* | *b/a/c* |
| 47 |  | 1.49 | 41.71 | 12.7 | 68.8 | 25.0 | 37.1 | 261.5 | 78.8 | 30.7 | *b* | *c/c/a* | *b/a/c* |
| 48 |  | 1.41 | 43.18 | 13.1 | 67.7 | 25.2 | 38.6 | 262.8 | 77.0 | 26.2 | *a* | *c/c/a* | *b/a/c* |
| 49 |  | 1.96 | 43.46 | 12.1 | 69.0 | 22.7 | 33.0 | 218.5 | 77.6 | 22.6 | *a* | *c/c/a* | *e/d/g* |
| 50 |  | 1.75 | 44.83 | 12.7 | 67.8 | 24.8 | 35.8 | 259.4 | 78.7 | 37.7 | *b* | *c/c/a* | *e/d/g* |
| 51 |  | 1.51 | 47.04 | 12.6 | 68.8 | 24.8 | 35.6 | 253.3 | 77.0 | 34.5 | *b* | *c/c/a* | *b/a/g* |
| 52 |  | 1.78 | 44.43 | 12.4 | 69.2 | 24.2 | 33.9 | 239.8 | 78.8 | 25.3 | *a* | *c/c/a* | *b/a/g* |
| 53 |  | 1.78 | 43.25 | 12.6 | 68.3 | 24.2 | 35.6 | 252.9 | 77.6 | 33.5 | *b* | *c/c/a* | *b/a/g* |
| 54 |  | 1.46 | 41.35 | 13.1 | 68.3 | 25.3 | 39.6 | 268.8 | 77.4 | 31.1 | *b* | *c/c/a* | *b/d/g* |
| 55 |  | 1.56 | 44.55 | 12.2 | 69.9 | 23.9 | 33.3 | 237.4 | 77.8 | 29.9 | *a* | *c/c/a* | *e/a/c* |
| 56 |  | 1.42 | 43.64 | 12.9 | 68.7 | 25.1 | 39.3 | 259.4 | 75.9 | 36.4 | *b* | *c/c/a* | *e/a/c* |
| 57 |  | 1.70 | 43.54 | 13.2 | 67.5 | 25.7 | 36.6 | 274.5 | 77.2 | 32.0 | *b* | *c/c/a* | *e/a/c* |
| 58 |  | 1.48 | 41.29 | 12.7 | 67.9 | 23.9 | 31.9 | 259.1 | 77.6 | 25.4 | *a* | *c/c/a* | *e/a/g* |
| 59 |  | 1.67 | 44.01 | 11.9 | 69.4 | 23.0 | 30.5 | 221.3 | 77.7 | 25.2 | *a* | *c/c/a* | *e/a/g* |
| 60 |  | 1.59 | 40.33 | 12.3 | 68.0 | 23.6 | 31.5 | 265.6 | 78.0 | 35.3 | *b* | *c/c/a* | *e/a/g* |
| R |  | 1.90 | 44.51 | 12.6 | 68.7 | 24.5 | 35.3 | 252.1 | 77.8 | 20.4 | *a* | *c/c/a* | *e/d/g* |
| F |  | 1.54 | 43.33 | 12.6 | 68.9 | 24.9 | 34.8 | 259.2 | 79.0 | 32.2 | *b* | *c/b/d* | *b/a/c* |
| Mean | | 1.60 | 42.51 | 12.7 | 68.4 | 24.7 | 35.4 | 259.2 | 77.9 | 30.0 |  |  |  |

R-Rysa

F-Finezja
