# Supplementary material for: Interactions between Glu-1 and Glu-3 loci and associations of selected molecular markers with quality traits in winter wheat (Triticum aestivum L.) DH lines
Source: J Appl Genet. 2016 Aug 8;58(1):37–48. doi: 10.1007/s13353-016-0362-5 (PMC5243893; doi:10.1007/s13353-016-0362-5)
Supplement: Supplementary file 2 — (DOCX 65 kb) [file 13353_2016_362_MOESM2_ESM.docx]

Interactions between Glu-1 and Glu-3 loci and associations of selected molecular markers with quality traits in winter wheat (*Triticum aestivum* L.) DH lines

Krystkowiak Karolina*, Langner Monika*, Adamski Tadeusz, Salmanowicz Bolesław, Kaczmarek Zygmunt, Krajewski Paweł, Surma Maria

Institute of Plant Genetics, Polish Academy of Sciences, Strzeszyńska 34, 60-479 Poznań, Poland

* E-mail: kkry@igr.poznan.pl

[mdyl@igr.poznan.pl](mailto:mdyl@igr.poznan.pl)

ESM_2. QTL identified in Rysa x Finezja population for analysed traits

| Trait | Marker | Chromosome | P-value for mean additive effect | P-value for interaction with years | Additive effect^a)^ | | | | S.e. of additive effect |
| --- | --- | --- | --- | --- | --- | --- | --- | --- | --- |
|  |  |  |  |  | 2010 | 2011 | 2012 | 2013 |  |
| GY | Xwmc312 | 1A | 0.958 | < 0.001 | 0.10 | -0.01 | -0.03 | -0.06 | 0.02 |
|  | Xwmc336 | 1D | 0.983 | < 0.001 | 0.09 | -0.01 | -0.08 | -0.01 | 0.02 |
|  | Xwmc407 | 2A | < 0.001 | 0.087 | 0.05 | 0.06 | 0.11 | 0.02 | 0.02 |
|  | Xgwm131 | 7B | 0.087 | < 0.001 | 0.03 | 0.01 | -0.12 | 0.00 | 0.02 |
|  | Xgwm635 | 7D | < 0.001 | 0.002 | -0.05 | -0.01 | -0.13 | -0.03 | 0.02 |
|  | Xbarc061 | 1B | 0.207 | < 0.001 | 0.01 | 0.02 | -0.11 | 0.02 | 0.02 |
|  | Xwmc419 | 1B | 0.952 | < 0.001 | 0.04 | 0.01 | -0.09 | 0.04 | 0.02 |
| TGW | Xbarc124 | 2A | < 0.001 | 0.382 | 0.16 | 0.29 | 0.60 | 0.52 | 0.20 |
|  | *GluB3* | 1B | < 0.001 | 0.698 | -0.25 | -0.45 | -0.57 | -0.35 | 0.20 |
|  | *GluD1* | 1D | < 0.001 | 0.549 | 0.69 | 0.58 | 0.55 | 0.30 | 0.20 |
|  | Xwmc112 | 2D | < 0.001 | 0.647 | 0.45 | 0.27 | 0.58 | 0.59 | 0.20 |
|  | Xwmc312 | 1A | < 0.001 | 0.001 | 0.22 | -0.78 | -0.78 | -0.36 | 0.21 |
|  | Xwmc36 | 1D | < 0.001 | 0.388 | 0.60 | 0.65 | 0.43 | 0.22 | 0.20 |
|  | Xwmc407 | 2A | < 0.001 | 0.090 | 0.37 | 0.40 | 0.85 | 0.98 | 0.21 |
|  | Xwmc732 | 1D | < 0.001 | 0.381 | 0.70 | 0.66 | 0.47 | 0.25 | 0.20 |
|  | Xwmc790 | 7A | < 0.001 | 0.193 | 0.67 | 0.64 | 1.15 | 0.69 | 0.19 |
|  | Xwmc93 | 1A | < 0.001 | 0.517 | 0.82 | 0.70 | 0.64 | 0.41 | 0.19 |
|  | Xgwm234 | 5A | < 0.001 | 0.400 | -0.28 | -0.47 | -0.37 | -0.75 | 0.20 |
|  | Xgwm55 | 6D | < 0.001 | 0.601 | -0.39 | -0.64 | -0.86 | -0.69 | 0.24 |
|  | Xbarc130 | 5D | 0.021 | < 0.001 | 0.53 | -0.59 | -0.24 | -0.63 | 0.20 |
|  | Xcfd48 | 1B | < 0.001 | 0.033 | -0.24 | -0.78 | -0.99 | -0.31 | 0.21 |
|  | Xgwm63 | 7A | < 0.001 | 0.020 | 0.22 | 0.65 | 1.08 | 0.69 | 0.19 |
|  | Xgpw315 | 1D | < 0.001 | 0.438 | 0.62 | 0.48 | 0.47 | 0.18 | 0.20 |
|  | Xgwm550 | 1B | < 0.001 | 0.906 | -0.30 | -0.36 | -0.44 | -0.49 | 0.20 |
|  | Xwmc608 | 2D | < 0.001 | 0.262 | -0.72 | -0.56 | -0.18 | -0.42 | 0.20 |
|  | Xgwm160 | 4A | < 0.001 | 0.145 | -0.80 | -0.63 | -0.70 | -1.20 | 0.19 |
|  | Xgwm408 | 5B | < 0.001 | 0.034 | -1.12 | -0.33 | -0.82 | -0.73 | 0.19 |
| PC | *GluB1* | 1B | < 0.001 | < 0.001 | 0.40 | 0.10 | 0.04 | 0.00 | 0.06 |
|  | *GluD1* | 1D | < 0.001 | 0.006 | -0.37 | -0.15 | -0.09 | -0.17 | 0.06 |
|  | Xgwm273 | 1B | < 0.001 | < 0.001 | 0.15 | 0.10 | 0.33 | -0.04 | 0.06 |
|  | Xwmc134 | 1B | < 0.001 | < 0.001 | 0.50 | 0.17 | 0.09 | 0.10 | 0.06 |
|  | Xwmc36 | 1D | < 0.001 | 0.181 | -0.28 | -0.12 | -0.11 | -0.16 | 0.06 |
|  | Xwmc93 | 1A | < 0.001 | < 0.001 | -0.39 | -0.10 | -0.04 | -0.13 | 0.06 |
|  | gpw4002 | 1B | < 0.001 | < 0.001 | 0.42 | 0.16 | 0.11 | 0.03 | 0.06 |
|  | Xgwm131 | 7B | < 0.001 | 0.555 | 0.21 | 0.12 | 0.14 | 0.09 | 0.06 |
|  | Xgwm106 | 1D | < 0.001 | 0.207 | 0.12 | 0.09 | 0.25 | 0.09 | 0.06 |
|  | Xgdm126 | 5A | < 0.001 | 0.914 | -0.17 | -0.14 | -0.13 | -0.19 | 0.06 |
|  | Xbarc130 | 5D | < 0.001 | 0.029 | -0.39 | -0.26 | -0.19 | -0.16 | 0.06 |
|  | Xgpw315 | 1D | < 0.001 | 0.295 | -0.23 | -0.14 | -0.06 | -0.14 | 0.06 |
|  | Xwmc527 | 3A | < 0.001 | 0.029 | 0.14 | 0.15 | 0.29 | 0.03 | 0.06 |
|  | Xwmc474 | 2A | < 0.001 | 0.001 | 0.38 | 0.23 | 0.09 | 0.07 | 0.06 |
|  | Xwmc344 | 2B | < 0.001 | 0.001 | 0.41 | 0.20 | 0.12 | 0.09 | 0.06 |
|  | Xwmc517 | 7B | < 0.001 | 0.111 | 0.27 | 0.15 | 0.18 | 0.06 | 0.06 |
| SC | *GluB3* | 1B | < 0.001 | 0.751 | -0.19 | -0.10 | -0.20 | -0.13 | 0.07 |
|  | *GluD1* | 1D | < 0.001 | 0.075 | 0.33 | 0.15 | 0.09 | 0.26 | 0.07 |
|  | Xgwm136 | 1A | < 0.001 | 0.504 | -0.25 | -0.12 | -0.11 | -0.14 | 0.07 |
|  | Xwmc134 | 1B | < 0.001 | < 0.001 | -0.47 | -0.12 | -0.05 | -0.20 | 0.07 |
|  | Xwmc36 | 1D | < 0.001 | 0.217 | 0.24 | 0.10 | 0.06 | 0.20 | 0.07 |
|  | Xwmc93 | 1A | < 0.001 | 0.014 | 0.37 | 0.12 | 0.05 | 0.21 | 0.07 |
|  | Xgpw4002 | 1B | < 0.001 | 0.001 | -0.43 | -0.12 | -0.07 | -0.10 | 0.07 |
|  | Xgpw7059 | 1B | < 0.001 | 0.916 | -0.12 | -0.20 | -0.14 | -0.15 | 0.08 |
|  | Xgwm234 | 5A | < 0.001 | 0.584 | -0.14 | -0.08 | -0.19 | -0.22 | 0.08 |
|  | Xbarc130 | 5D | < 0.001 | 0.022 | 0.49 | 0.23 | 0.26 | 0.22 | 0.07 |
|  | Xgpw315 | 1D | < 0.001 | 0.139 | 0.26 | 0.10 | 0.03 | 0.17 | 0.07 |
|  | Xwmc474 | 2A | < 0.001 | 0.009 | -0.36 | -0.12 | -0.04 | -0.11 | 0.07 |
|  | Xwmc344 | 2B | < 0.001 | < 0.001 | -0.45 | -0.15 | -0.05 | -0.13 | 0.07 |
| WG | *GluB1* | 1B | < 0.001 | < 0.001 | 0.92 | 0.24 | 0.02 | 0.00 | 0.15 |
|  | *GluD1* | 1D | < 0.001 | 0.057 | -0.84 | -0.35 | -0.34 | -0.56 | 0.15 |
|  | Xgwm273 | 1B | < 0.001 | < 0.001 | 0.34 | 0.35 | 0.79 | -0.17 | 0.15 |
|  | Xwmc134 | 1B | < 0.001 | < 0.001 | 1.14 | 0.36 | 0.16 | 0.22 | 0.15 |
|  | Xwmc36 | 1D | < 0.001 | 0.342 | -0.65 | -0.27 | -0.40 | -0.51 | 0.15 |
|  | Xwmc93 | 1A | < 0.001 | 0.002 | -0.91 | -0.25 | -0.21 | -0.37 | 0.15 |
|  | Xgpw4002 | 1B | < 0.001 | < 0.001 | 0.90 | 0.37 | 0.16 | 0.05 | 0.15 |
|  | Xgpw7062 | 1A | < 0.001 | 0.528 | -0.40 | -0.11 | -0.40 | -0.31 | 0.16 |
|  | Xgwm131 | 7B | < 0.001 | 0.511 | 0.50 | 0.36 | 0.25 | 0.18 | 0.16 |
|  | Xgwm106 | 1D | < 0.001 | 0.101 | 0.34 | 0.27 | 0.72 | 0.24 | 0.16 |
|  | Xgdm126 | 5A | < 0.001 | 0.905 | -0.37 | -0.31 | -0.40 | -0.47 | 0.15 |
|  | Xbarc130 | 5D | < 0.001 | 0.117 | -0.94 | -0.65 | -0.58 | -0.45 | 0.15 |
|  | Xgwm642 | 1D | < 0.001 | 0.905 | -0.39 | -0.24 | -0.33 | -0.36 | 0.15 |
|  | Xgpw315 | 1D | < 0.001 | 0.703 | -0.47 | -0.32 | -0.26 | -0.46 | 0.15 |
|  | Xwmc527 | 3A | < 0.001 | 0.016 | 0.31 | 0.39 | 0.74 | 0.06 | 0.15 |
|  | Xwmc474 | 2A | < 0.001 | 0.004 | 0.89 | 0.56 | 0.25 | 0.22 | 0.15 |
|  | Xwmc344 | 2B | < 0.001 | 0.002 | 0.99 | 0.44 | 0.30 | 0.26 | 0.15 |
|  | Xwmc517 | 7B | < 0.001 | 0.124 | 0.68 | 0.40 | 0.42 | 0.16 | 0.15 |
| ZS | *GluB1* | 1B | < 0.001 | < 0.001 | 2.91 | 0.33 | 0.19 | 0.02 | 0.37 |
|  | *GluD1* | 1D | < 0.001 | < 0.001 | -2.33 | -0.55 | -0.19 | -1.01 | 0.38 |
|  | Xgwm273 | 1B | < 0.001 | < 0.001 | 1.12 | 0.44 | 2.47 | -0.09 | 0.38 |
|  | Xwmc134 | 1B | < 0.001 | < 0.001 | 3.39 | 0.58 | 0.28 | 0.47 | 0.37 |
|  | Xwmc36 | 1D | < 0.001 | 0.039 | -1.68 | -0.45 | -0.24 | -0.98 | 0.38 |
|  | Xwmc93 | 1A | < 0.001 | < 0.001 | -2.61 | -0.38 | 0.06 | -0.78 | 0.37 |
|  | Xgpw4002 | 1B | < 0.001 | < 0.001 | 3.01 | 0.53 | 0.70 | 0.11 | 0.37 |
|  | Xgwm131 | 7B | < 0.001 | 0.044 | 1.92 | 0.50 | 0.92 | 0.65 | 0.39 |
|  | Xgwm106 | 1D | < 0.001 | 0.027 | 1.10 | 0.41 | 1.99 | 0.72 | 0.39 |
|  | Xbarc061 | 1B | < 0.001 | < 0.001 | 0.98 | 0.46 | 2.07 | -0.44 | 0.39 |
|  | Xbarc130 | 5D | < 0.001 | 0.002 | -2.90 | -1.02 | -1.57 | -1.24 | 0.37 |
|  | Xwmc527 | 3A | < 0.001 | 0.014 | 0.78 | 0.67 | 2.00 | 0.37 | 0.38 |
|  | Xwmc474 | 2A | < 0.001 | < 0.001 | 2.54 | 0.93 | 0.39 | 0.49 | 0.38 |
|  | Xwmc344 | 2B | < 0.001 | < 0.001 | 2.87 | 0.80 | 0.63 | 0.62 | 0.37 |
|  | Xgdm36 | 1B | < 0.001 | < 0.001 | 1.35 | 0.23 | 1.92 | -0.52 | 0.38 |
|  | Xwmc517 | 7B | < 0.001 | 0.005 | 2.13 | 0.57 | 1.29 | 0.41 | 0.38 |
|  | Xwmc419 | 1B | < 0.001 | < 0.001 | 1.01 | 0.39 | 1.96 | -0.35 | 0.38 |
| APW | *GluD1* | 1D | < 0.001 | 0.047 | -11.87 | -4.82 | -4.79 | -7.88 | 2.05 |
|  | Xwmc134 | 1B | < 0.001 | < 0.001 | 13.54 | 4.29 | 0.05 | 2.06 | 2.11 |
|  | Xwmc36 | 1D | < 0.001 | 0.289 | -8.58 | -3.25 | -5.50 | -7.44 | 2.09 |
|  | Xwmc93 | 1A | < 0.001 | 0.003 | -12.29 | -3.68 | -2.58 | -4.99 | 2.05 |
|  | Xgwm234 | 5A | < 0.001 | 0.775 | 3.37 | 3.64 | 5.79 | 5.86 | 2.21 |
|  | Xgwm95 | 2A | < 0.001 | 0.281 | 4.30 | 2.87 | 8.32 | 3.74 | 2.13 |
|  | Xgwm106 | 1D | < 0.001 | 0.206 | 3.64 | 3.88 | 8.59 | 2.56 | 2.16 |
|  | Xgdm126 | 5A | < 0.001 | 0.774 | -5.32 | -3.20 | -5.98 | -5.90 | 2.13 |
|  | Xbarc130 | 5D | < 0.001 | 0.265 | -13.54 | -9.40 | -8.68 | -8.65 | 2.03 |
|  | Xgpw315 | 1D | < 0.001 | 0.361 | -8.15 | -3.59 | -3.80 | -6.19 | 2.09 |
|  | Xwmc474 | 2A | < 0.001 | 0.011 | 9.51 | 7.67 | 1.38 | 2.07 | 2.08 |
|  | Xwmc344 | 2B | < 0.001 | 0.002 | 12.32 | 6.21 | 2.41 | 2.73 | 2.07 |
| HW | Xgpw2276 | 1A | 0.017 | < 0.001 | 0.21 | -0.37 | -0.40 | 0.05 | 0.11 |
|  | Xwmc751 | 3B | < 0.001 | 0.370 | -0.46 | -0.35 | -0.40 | -0.19 | 0.11 |
| GH | *GluD1* | 1D | < 0.001 | 0.003 | -3.01 | -1.12 | -0.31 | -1.68 | 0.53 |
|  | Xgwm111 | 7D | < 0.001 | 0.979 | 1.78 | 1.63 | 1.97 | 1.73 | 0.55 |
|  | Xwmc134 | 1B | 0.006 | < 0.001 | 3.20 | 0.66 | -0.77 | -0.07 | 0.55 |
|  | Xwmc36 | 1D | < 0.001 | 0.087 | -2.16 | -0.95 | -0.23 | -1.34 | 0.54 |
|  | Xwmc667 | 2A | < 0.001 | 0.547 | -1.91 | -1.25 | -0.77 | -1.20 | 0.56 |
|  | Xwmc818 | 1A | < 0.001 | 0.533 | -1.97 | -0.91 | -1.15 | -1.08 | 0.55 |
|  | Xwmc93 | 1A | < 0.001 | < 0.001 | -3.13 | -0.61 | 0.24 | -0.84 | 0.54 |
|  | Xgwm95 | 2A | < 0.001 | 0.530 | 1.38 | 0.44 | 1.35 | 1.45 | 0.56 |
|  | Xgwm106 | 1D | < 0.001 | 0.910 | 1.58 | 1.14 | 1.64 | 1.31 | 0.55 |
|  | Xbarc130 | 5D | < 0.001 | 0.001 | -5.22 | -2.70 | -2.69 | -3.42 | 0.49 |
|  | Xgpw315 | 1D | < 0.001 | 0.157 | -2.14 | -1.07 | -0.56 | -1.77 | 0.54 |
|  | Xwmc474 | 2A | < 0.001 | 0.003 | 2.50 | 1.68 | -0.18 | 0.66 | 0.54 |
|  | Xwmc344 | 2B | < 0.001 | 0.001 | 2.98 | 1.41 | -0.08 | 0.62 | 0.53 |
|  | Xwmc517 | 7B | < 0.001 | 0.171 | 2.04 | 1.42 | 0.43 | 0.82 | 0.54 |
|  | Xgwm160 | 4A | < 0.001 | 0.785 | -1.48 | -0.74 | -1.28 | -0.97 | 0.55 |

a) Additive effects: negative—alleles increasing trait value from Finezja. positive—alleles increasing trait value from Rysa
